# Resistance training decreases 24-hour blood pressure in women with metabolic syndrome

**DOI:** 10.1186/1758-5996-5-27

**Published:** 2013-05-27

**Authors:** Ramires Alsamir Tibana, Guilherme Borges Pereira, Jéssica Cardoso de Souza, Vitor Tajra, Denis Cesar Leite Vieira, Carmen Silvia Grubert Campbell, Claudia Regina Cavaglieri, Jonato Prestes

**Affiliations:** 1 Graduate Program on Physical Education and Health, Catholic University of Brasilia (UCB), Brasilia, Brazil; 2 Federal University of Maranhão (UFM), Maranhão, Brazil; 3 School of Physical Education, State University of Campinas, Campinas, Brazil

**Keywords:** Resistance training, Metabolic syndrome, Blood pressure

## Abstract

**Background:**

The purpose of this study was to verify the effects of eight weeks of resistance training (RT) on 24 hour blood pressure (BP) in patients with and without metabolic syndrome (MetS).

**Methods:**

Seventeen women volunteered to participate in this study, 9 with MetS (37.0 ± 8.7 yrs; body mass 77.3 ± 9.7 kg; body mass index 30.3 ± 4.2 kg · m^-2^) and 8 without MetS (35.1 ± 7.2 yrs; body mass 61.3 ± 8.1 kg; body mass index 24.2 ± 2.5 kg · m^-2^). Individuals were subjected to eight weeks (3 times/week) of whole body RT comprised of one exercise for each main muscle group with three sets of 8–12 repetitions of each subject’s maximal load . A rest interval of one minute was allowed between sets and exercises. Twenty-four hour BP was measured by ambulatory blood pressure monitoring.

**Results:**

Mean and diastolic night-time BP decreased (−3.9 mmHg, p = 0.04; -5.5 mmHg, p = 0.03, respectively) after eight weeks of training in MetS patients. This decrease was observed at 11:00 pm, 02:00 am (only diastolic), 07:00 am, and 6:00 pm. There was no training effect on BP in women without MetS.

**Conclusions:**

Considering the elevation of BP as a contributor to the pathogenesis of MetS, and also to the increase of cardiovascular risk, this study supports RT as a non-pharmacological therapy in the management of BP control for MetS.

## Background

Cardiovascular disease has been identified as a leading cause of death in the United States with medical costs and productivity losses reaching approximately $450 billion. Additionally, more than 2 million Americans have a heart attack or stroke each year, and more than 800,000 of them die every year as consequence of cardiovascular problems [1].

Metabolic syndrome (MetS) is characterized by the grouping of several cardiovascular risk factors such as central obesity, dyslipidemia, insulin resistance, elevated glucose plasma levels and elevated blood pressure (BP) [2]. Moreover, it has been shown that the relative risk of cardiovascular disease and death in individuals with MetS is 1.78 higher than in individuals without MetS [3]. The prevalence of MetS has increased in developing countries in recent decades, while alarming data revealed a 32.0% prevalence of MetS in the central region of Brazil [4]. Thus, the decrease of atherogenic dyslipidemia, prothrombotic state and BP is important to reduce the risk for cardiovascular diseases. Therefore, this reinforces the importance of developing effective and affordable strategies such as exercise programs to prevent and treat cardiovascular risks [5].

Current recommendations for the treatment of MetS and the primary prevention of cardiovascular diseases encourage the use of therapeutic lifestyle changes such as increasing physical activity, reducing dietary intake, and achieving weight loss [6-8]. Regarding physical activity interventions, few studies have reported the effectiveness of exercise programs alone for multiple cardiovascular risk factors [8-10]. Smutok *et al*., [11] compared the effects of 20 weeks of resistance training (RT) to aerobic training, and observed that both interventions resulted in increased glucose tolerance and insulin sensitivity, with no changes in lipoprotein lipids and BP. Katzel *et al*., [12] found that aerobic exercise alone did not improve the lipid profile and BP. However, the HERITAGE Family Study revealed that 20 weeks of aerobic exercise training in patients with the MetS resulted in a decrease of 38% clinical BP [11].

To have clinical relevance, the decrease in BP should be maintained for many hours and sustained during daily activities [13]. Studies with RT revealed a significant post-exercise hypotensive effect, although most studies were limited to the analysis of BP during a specific period after exercise under laboratorial conditions, which is called clinical BP [14]. Thus, the use of ambulatory blood pressure (ABP) monitoring, which provides a spectrum of the 24 h hemodynamic load, has been recently expanded [15]. A recent meta-analysis suggests that moderate-intensity RT can reduce systolic blood pressure (SBP) and diastolic blood pressure (DBP), increase in VO_2_peak, and decrease body fat and plasma triglycerides, both in normotensive and pre-hypertensive subjects [16]. However, the evidence on the chronic effects of dynamic RT on ABP monitoring remains understudied in patients with MetS.

Existing data regarding 24 h BP and the post-exercise hypotensive effect are both scarce and controversial. Considering this, the aim of the present study was to verify the effects of eight weeks of RT on 24 h BP in patients with and without MetS. The initial hypothesis was that the RT program would be effective in decreasing BP during sleeping hours and daily activities in patients with MetS.

## Methods

### Subjects

Twenty-five middle-aged women volunteered to participate in the study. However, because of time constraints, eight subjects (three from the MetS group and five from the group without MetS) dropped out or did not complete the entire protocol. Therefore, nine patients with MetS and eight without MetS successfully completed the study. The inclusion criteria for participation in the study included age above 18 years, no RT experience, being free of clinical problems that could be aggravated by the protocol and ability to attend more than 85% of the training sessions. Exclusion criteria for the group without MetS included the chronic use of any medication and hypertension (SBP >140 mmHg and DBP > 90 mmHg). Additional exclusion criteria for women with and without MetS included regular exercise, subjects with physical disabilities, diagnosis of diabetes, cardiovascular diseases, musculoskeletal disease and recent smoking or drug/alcohol abuse. Resting 12-lead electrocardiogram recordings obtained from all subjects before participation in this study showed normal electrocardiographic patterns. Sedentary state was defined by the International physical activity questionnaire (IPAQ). The study protocol was approved by the Catholic University of Brasilia Ethics Committee (protocol #376/2010), and patients gave written, informed consent.

Metabolic syndrome was defined according to the American Heart Association and National Heart, Lung, and Blood Institute scientific statement [17]. The criteria for diagnosis of the MetS was the presence of ≥3 of the following characteristics: elevated waist circumference (≥88 cm), elevated triglycerides (TG) (≥150 mg/dl) or receiving drug treatment for elevated TG, reduced HDL cholesterol (≤50 mg/dl) or receiving medication for reduced HDL cholesterol, elevated BP (≥130 mm Hg SBP or ≥85 mm Hg DBP) or receiving antihypertensive drug treatment with a history of hypertension, elevated fasting glucose (≥100 mg/dl) or receiving medication for elevated glucose. A modified z score was calculated for each variable using individual subject data using the Adult Treatment Panel (ATP) III criteria. The equations used to calculate the MetS z score were as follows: {z score = [(50 - HDL)/11.8] + [(TG −150)/66.2] + [(fasting blood glucose - 110)/10.4] + [(waist circumference - 88)/9.2] + [(mean arterial pressure - 100)/8.7]/100}.

### Study design

The study investigated the effects of eight weeks of RT on 24-hour BP in women with and without MetS. The RT program was performed on three non-consecutive days of the week (Monday/Wednesday/Friday) and was comprised of three sets of 8–12 repetitions maximum (RM) of 12 exercises, and 1-minute rest intervals between sets and exercises performed. All testing and training sessions were conducted between 08:00–09:00 pm. Before training and four days after the end of the RT all subjects underwent 24 h BP monitoring (Dyna-MAPA®, Cardios, Brazil). All subjects received instructions about the technique and position of the cuff during the 24 h BP monitoring. In addition, the subjects were encouraged to avoid smoking, alcohol, caffeine, unusual physical activity and to maintain their usual diet consumption (this was confirmed by a dietary recall follow-up). Individuals were asked to go to bed at 11:00 pm and awaken at 06:00 am on recording days.

### Biochemical parameters

Participants reported to the laboratory between 08:00–10:00 am, after an overnight fast, for blood withdrawal from the antecubital vein. Triglycerides, HDL-cholesterol and glucose were measured by the following methods: enzymatic CHOP-POD, homogeneous HDL-cholesterol and Hexokinase, respectively.

### Anthropometric and body composition evaluation

Height and weight were measured for the calculation of the body mass index (BMI; body mass in kg divided by square of height in meters). All circumferences were obtained using a non-elastic tape; measurements were obtained in triplicate and then averaged. Neck circumference was obtained with the subject sitting with the head in the Frankfort horizontal plane position. A measuring tape was applied around the neck inferior to the laryngeal prominence and perpendicular to the long axis of the neck, while the minimal circumference was measured and recorded to the nearest 0.1 cm [18]. Waist circumference was measured at the midway level between the lower rib margin and the iliac crest [18]. The body fat percentage was determined by the Jackson and Pollock equation [19].

### Twenty-four hour blood pressure monitoring

Twenty-four hour BP was measured in the non-dominant arm with an oscillometric monitor (Dyna-MAPA®, Cardios, Brazil) in accordance with manufacturer’s instructions before the initiation of the training program and four days after the eight weeks of RT were finished. The monitor was programmed to perform measures every 15 minutes during daytime and every 30 minutes during the sleeping hours. All ambulatory measures of BP were performed during weekdays (i.e. Monday to Friday), and initiated between 9:00–10:00 pm. All participants were advised to maintain their habitual activities, refrain from programmed exercise for at least 72 h before the measures, and to stop any activity and relax the arm during each measurement. Data were calculated and analyzed as follows: mean of all measurements during the 24 h period, and mean of all measures performed during daytime and sleeping hours. The BP measurements were considered invalid for analysis if >30% of the measurements were missing, if data were lacking for an interval of >2 h, or if the sleeping hours period was <6 or >12 h [20]. Individuals were advised to control their liquid ingestion before data acquisition. Additionally, the cuff size was adapted to the circumference of the arm of each participant according to the manufacturer’s recommendations.

### Resistance training program

Before initiation of the experimental sessions, the volunteers completed two weeks of familiarization prior to testing [21]. During the familiarization weeks, individuals were advised regarding the execution of proper technique, and completed 3 sessions/week, with one exercise for each main muscle group (same exercises of the RT). Individuals performed 3 sets of 10–12 submaximal repetitions at 60% of estimated 10RM. Machines were from JOHNSON – USA.

**Figure 1:**
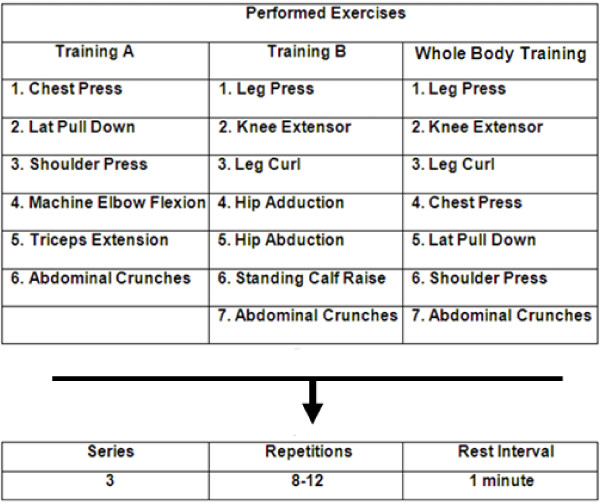
**Division of the RT sessions throughout the study.** For all exercises, 3 sets with 8–12 repetitions maximum were performed with a 1-minute rest interval between each set and exercise.

The RT program consisted of 3 sessions/week for eight weeks. All training sessions were carefully supervised by three physical education professors (ratio of supervision 1:2 – 1 professor for 2 participants). Participants were required to complete at least 85% of the exercise sessions. No major complications or cardiac events occurred during the study period. Figure 1 shows the exercise order that was strictly followed by both groups. The RT was divided into A (Monday) and B (Wednesday) and whole body (Friday) regiments. For both groups, abdominal crunches (three sets of 15 repetitions in all sessions) were included. For all listed exercises, three sets with 8–12 RM were performed [22], with a one-minute rest interval between each set and exercise. The average duration to complete one repetition was 3–4 s (both concentric and eccentric phases of the movement). The total exercise time was ≈ 40 - 50 min for all regimens. The number of repetitions and the loads used for each exercise session were recorded. The loads were updated when necessary to keep the number of repetitions within the same range of RM and to provide a progressive overload. Additionally, the participants were instructed to maintain their normal food intake during the research and correct breathing patterns were instructed to avoid Valsalva maneuver.

### Statistical analyses

The normal distribution of the data was checked using the Shapiro-Wilk normality test and the Mauchly homoscedasticity test. The pre-intervention variables of the two groups were compared by using unpaired Student’s *t*-test. The effects of the RT on the experimental groups were assessed by a dependent Student’s *t*-test. Considering a power (1 – β) of 0.80 and an alpha error of 0.05, the sample size used in this research allowed identifying a moderate effect size. P < 0.05 was accepted as significant, and data are reported as means ± SD. Data were analyzed using the Statistical Package for Social Sciences (SPSS, v.19, Chicago, IL).

## Results

General subjects characteristics are presented in Table 1. The MetS group presented higher values for body mass, lean body mass, fat body mass, body mass index, percentage body fat, neck circumference, waist and hip circumference and triglycerides as compared with the group without MetS.

**Table 1:** Baseline characteristics of subjects

	**MetS**	**Without MetS**	**P value**
	(**n** = **9**)	(**n** = **8**)	
***Anthropometric parameters***			
Age (years)	37.0 ± 8.7	35.1 ± 7.2	0.98
Body Mass (kg)	77.3 ± 9.7*	58.2 ± 6.2	0.002
Height (m)	1.60 ± 0.08	1.59 ± 0.05	0.51
Neck Circumference (cm)	35.7 ± 1.5*	33.1 ± 1.7	0.002
Waist Circumference (cm)	90.9 ± 9.2*	79.0 ± 8.7	0.009
Hip Circumference (cm)	107.1 ± 7.7*	98.0 ± 6.3	0.01
***Body composition parameters***			
Body Mass Index (kg · m^-2^)	30.3 ± 4.2*	24.2 ± 2.5	0.001
Body Fat (%)	37.1 ± 3.1*	28.3 ± 3.9	<0.001
Fat Body Mass (kg)	28.9 ± 5.6*	16.6 ± 3.0	0.02
Lean Body Mass (kg)	48.7 ± 6.0*	41.6 ± 4.1	0.02
***Biochemical Parameters***			
Blood Glucose (mg · dL)	95.2 ± 22.2	85.6 ± 4.5	0.33
Triglycerides (mg · dL)†	115.0 ± 37.4*	62.7 ± 20.8	0.006
HDL-C (mg · dL)	41.2 ± 8.5*	59.0 ± 12.3	0.05
*Z*-*Score MetS*	1.36 ± 0.13*	1.16 ± 0.06	0.001
***Hemodynamic variables***			
Systolic Blood Pressure (mmHg)	131.8 ± 16.1*	108.6 ± 6.2	0.002
Diastolic Blood Pressure (mmHg)	84.1 ± 11.3*	74.0 ± 6.1	0.03
Mean Blood Pressure (mmHg)	100.1 ± 11.9*	85.5 ± 4.8	0.005
Heart Rate (bpm)	81.4 ± 16.9	76.0 ± 14.3	0.33

The data are presented as mean ± SD. *Significantly different between groups; ^†^Values expressed as median; *HDL* high density lipoprotein, *MetS* metabolic syndrome.

**Figure 2:**
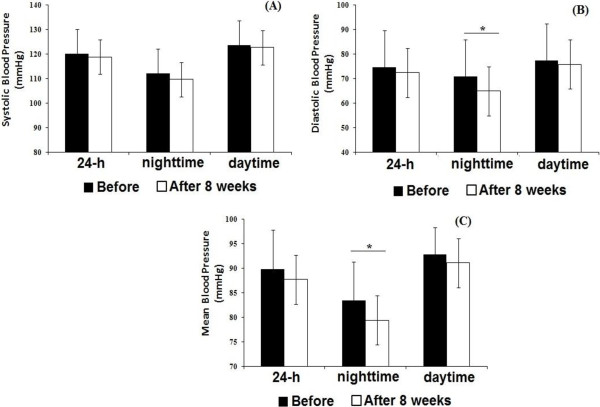
**Twenty four hour (24 h), daytime and nighttime ambulatory blood pressure monitoring**, **before and after eight weeks of resistance training in individuals with metabolic syndrome.** Systolic blood pressure (panel **A**); diastolic blood pressure (panel **B**); mean blood pressure (panel **C**). *different from pre-training (p < 0.05). Valeus are presented as mean ± SD.

**Figure 3:**
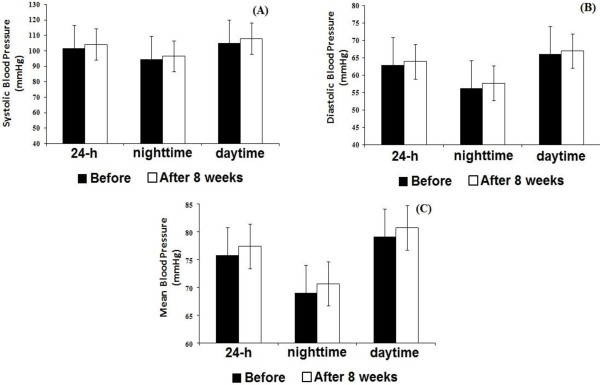
**Twenty four hour** (**24 h**), **daytime and nighttime ambulatory blood pressure monitoring**, **before and after eight weeks of resistance training in individuals without metabolic syndrome.** Systolic blood pressure (panel **A**); diastolic blood pressure (panel **B**); mean blood pressure (panel **C**). Values are presented as mean ± SD.

Figures 2 and 3 present mean values of 24 h, daytime and nighttime periods of BP monitoring before and after 8 weeks of RT in MetS and without MetS groups, respectively. Mean blood pressure (MBP) and DBP was decreased during nighttime in MetS group after the RT program (Figure 2). No statistically significant differences were observed in the group without MetS for 24 h, daytime or nighttime analyses after the RT program (Figure 3).

**Figure 4:**
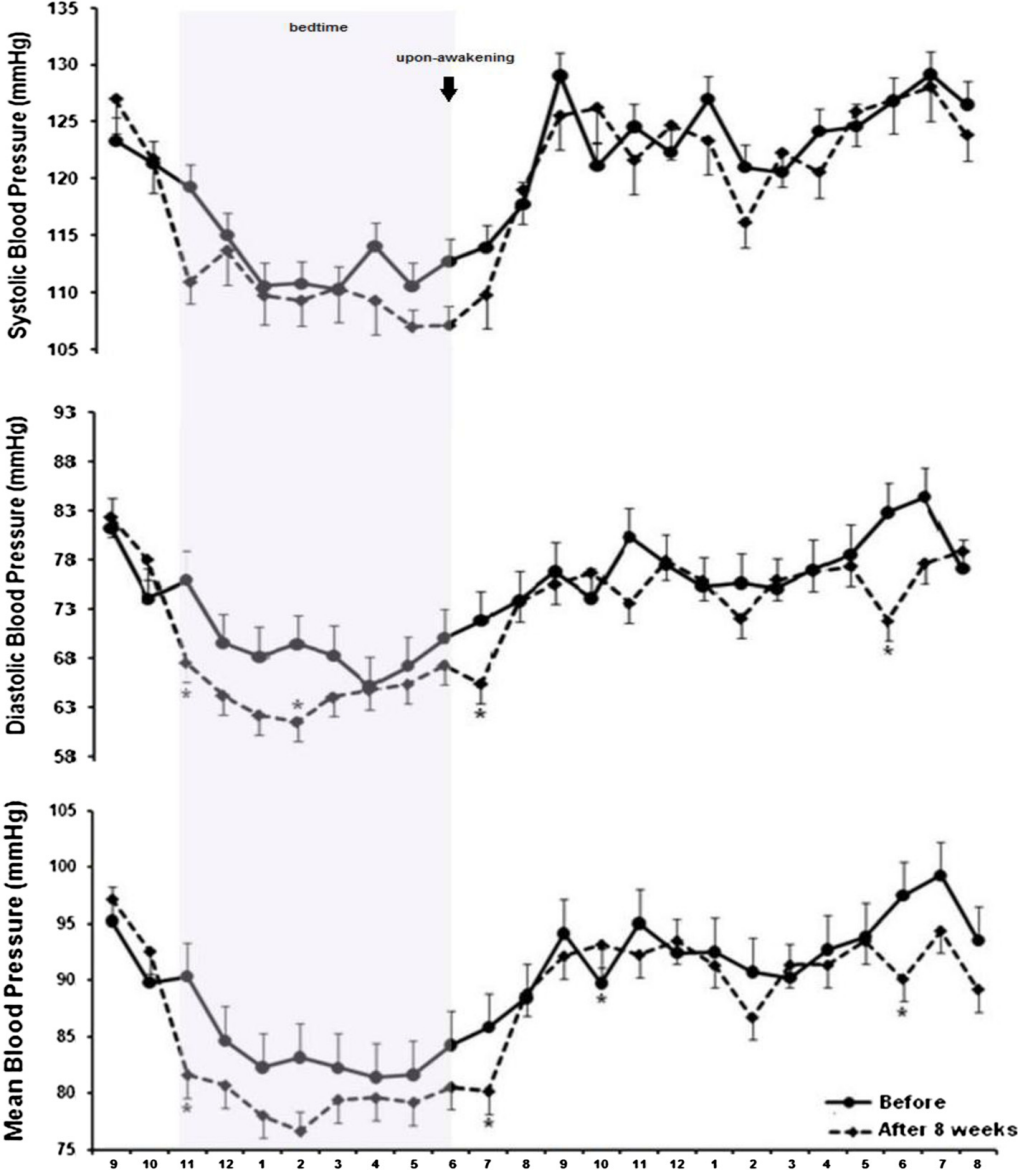
**Twenty four hour blood pressure monitoring before and after eight weeks of resistance training in the metabolic syndrome group.** *different from pre-training (p ≤ 0.05). Values are presented as mean ± SD.

**Figure 5:**
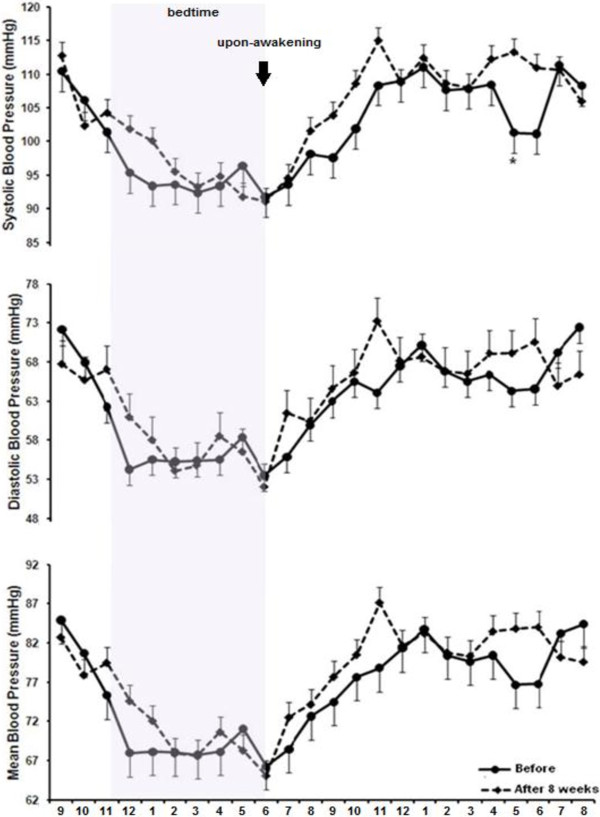
**Twenty four hour blood pressure monitoring before and after eight weeks of resistance training in the group without metabolic syndrome.** Values are presented as mean ± SD.

Figure 4 shows 24 h BP measurement for SBP, DBP and MBP before (pre-intervention) and after 8 weeks of RT in the MetS group. DBP values decreased at 11:00 pm (tendency from 75.5 to 67; -8.5 mmHg; p = 0.06 [−0.57,17.35 - CI]), 02:00 am (from 70 to 61; -9 mmHg; p = 0.04 [−13.45, -0.04 - CI]), 07:00 am (from 71 to 65; -6 mmHg; p = 0.03 [0.71, 12.51 - CI]) and 6:00 pm (from 81 to 71; -10 mmHg; p = 0.02 [−2.22, 19.84 - CI]) after eight weeks of training. MBP values decreased at 11:00 pm (from 91 to 81; -10 mmHg; p = 0.04 [0.39, 17.34 - CI]), 7:00 am (tendency from 85.2 to 80.2; -5.6 mmHg; p = 0.06; [−12.02, 0.57 - CI]), and 6 pm (from 97.5 to 90.2; -7.3 mmHg; p = 0.03; [−13.87, -0.80 - CI]) after the RT program. There was no statistically significant difference for SBP. No statistically significant alterations on MBP, DBP and SBP were observed after eight weeks of RT in the group without MetS (Figure 5).

## Discussion

To the best of our knowledge, this is the first study to evaluate the effects of eight weeks of RT on 24 h BP in individuals with MetS. Our initial hypothesis has been partially confirmed, since MBP and DBP decreased in the MetS group after the RT program, while SBP values remained stable. The decrease mainly occurred at 11:00 pm, 02:00 am, 07:00 am, and 6:00 pm. Furthermore, there was no effect of RT on 24 h BP in women without MetS.

In daily practice, RT is employed to increase muscle strength parameters and improve metabolic profile parameters, which is favorable to the maintenance of functional capacity, prevention of sarcopenia, osteoporosis, obesity, hypertension and MetS [23-25]. However, contrary to endurance training, evidence for a BP-lowering effect of RT remains scarce and much less compelling. A meta-analysis from Cornelissen and Fagard [25] showed that RT reduces systolic and diastolic clinical BP by 3.2 and 3.5 mmHg, respectively in normotensive and hypertensive adults. Although relevant, these results should be analyzed with caution, because BP measurements were mainly collected at a specific period after training (most studies up to 60 minutes after training). Moreover, clinical relevance of BP decrease after a single or chronic RT should be maintained for many hours during day and nighttime periods [13,15,26]. The importance of chronic BP reductions and maintenance during the day can be estimated from large, prospective intervention studies investigating morbidity and mortality outcomes that suggest that small reductions in resting SBP and DBP of 3 mmHg can reduce stroke by 8%, coronary heart disease risk by 5%, and all-cause mortality by 4% [26-29].

In the present study, women with MetS presented a night-time decrease of MBP (−6 to −10 mmHg) and DBP (−5.6 to −8.7 mmHg) mainly at 11:00 pm, 02:00 am (only diastolic), 07:00 am and 6:00 pm after eight weeks of RT. Most studies recommend 24 h BP as a better predictor of morbidity and mortality than clinical BP [15,26,30]. However, there is still debate on the relative importance of daytime and nighttime 24 h BP. Furthermore, Fagard *et al*., [29] demonstrated that nighttime BP is in general a better predictor of outcome than daytime pressure in hypertensive patients, and the night–day BP ratio predicts mortality, even after adjustment for 24 h BP [27,31]. The recent MAPEC (Ambulatory Blood Pressure Monitoring and Cardiovascular Events) study [32] revealed a significant relationship between decreasing BP during sleeping hours and reduced cardiovascular risk in subjects with normal or elevated BP, suggesting risk-reduction benefits even below the current diagnostic threshold of 120 mmHg. Thus, the decrease of BP during sleeping hours should be considered as a novel therapeutic target for reduction of cardiovascular risk [32].

In this sense, this reinforces the clinical relevance of the present results. The drop of BP during sleeping hours in women with MetS would be of great importance in preventing the development of hypertension and cardiovascular diseases [2,3]. Although the mechanisms associated with post-exercise hypotension have not been the focus of the present study, it is possible to speculate that several hemodynamic and neuro-humoral factors, such as decreased sympathetic activity, plasma catecholamine levels and vascular tone interacted to produce this circadian variation in BP [30]. Additional proposals include a decreased cardiac output and peripheral vascular resistance [33-35], higher activity of the plasma kallikrein system mediating nitric oxide release [36], and alterations in cerebral blood flow induced by exercise [34]. More studies will be necessary to adequately address the mechanisms responsible for the reduction of MBP and DBP after RT.

Although acute hypotensive effects of aerobic [37] and resistance exercise [38,39] has been widely shown in different populations, the chronic effects of RT on 24 h BP are controversial and scarce. Pescatello et al., [40] investigated the acute effects of 30 minutes of aerobic exercise (cycle ergometer) at 40% or 60% of VO_2_peak and found a non-significant drop of BP up to nine hours after exercise in men with MetS. Stensvold et al., [41] compared the effects of interval aerobic training, RT and aerobic + RT on risk factors of MetS and found a non-significant chronic decrease of BP in individuals with MetS. The difficulties in comparing the results from previously published studies with our results are that most studies with MetS and exercise were acute used limited time-course analyses of BP and different exercise protocols. To note, the RT used in the present study is practical and widely recommended, as we used one resistance exercise for each main muscle group, which reinforces the clinical relevance of the results.

The results of the present study showed no effect of RT on 24 h BP during daytime and nighttime analyses in women without MetS. The failure to observe chronic hypotensive effects of RT is in accordance with previous studies investigating 24 h BP in normotensive or mild hypertensive subjects [16,42].

Some limitations of the present study should be considered. The circadian pattern of BP has a strong genetic dependency in terms of daytime, the amplitude of variation, and peak time during a 24 h period. No mechanisms regarding the chronic decrease of BP were investigated, the number of participants in each group was small and the intervention lasted only eight weeks.

Furthermore, the use of 48 h BP monitoring instead of the more common 24 h could increase the reproducibility of the BP findings [42], and use of wrist actigraphy to precisely and individually determine the beginning and ending of the activity and sleep spans for each subject could enable the accurate calculation of the awake and sleeping BP means [26].

In conclusion, after 8 weeks of a resistance-training program, the mean blood pressure and diastolic blood pressure decreased during day and sleeping hours in women with metabolic syndrome. The preset results provide a possible non-pharmacological intervention strategy for decreasing sleep-time blood pressure that could be evaluated in patients by ambulatory monitoring. Future studies should be designed to reinforce our findings and evaluate the markers of cardiovascular morbidity and mortality. In addition, studies evaluating the effects of RT performed during different periods of the day (morning versus night) would improve the understanding regarding the circadian rhythm of blood pressure.

## Competing interests

This research received no specific grant from any funding agency in the public, commercial, or not-for-profit sectors. The authors declare no competing interests.

## Authors’ contributions

RAT, GBP, JCdeS, CRC and JP were responsible for concept and design, statistical expertise, data analysis and interpretation and helped to write the manuscript. DV, VT: CSGC were significant manuscript reviewers/revisers and were responsible for data analysis and interpretation. All authors have read and approved the manuscript for publication.
